# Synthesis of a new sulfadimidine Schiff base and their nano complexes as potential anti-COVID-19 and anti-cancer activity

**DOI:** 10.1038/s41598-023-28402-9

**Published:** 2023-01-27

**Authors:** Shimaa Hosny, Mona S. Ragab, Randa F. Abd El-Baki

**Affiliations:** 1 Department of Chemistry, Faculty of Science, New Valley University, Alkharga, 72511 Egypt; 2 Department of Chemistry, Faculty of Science, Cairo University, Giza, 12613 Egypt

**Keywords:** Coordination chemistry, Green chemistry

## Abstract

The primary objective of this study was to describe the cytotoxicity on HEPG-2 cells and to study the COVID‑19 activities of the novel H_2_L ligand and its Cr and Cu nano-complexes. As well as exploring the chemistry of the prepared nano-complexes. In this paper novel Schiff base, N-(4, 6-dimethyl pyrimidin-2-yl)-4-(((2-hydroxyl naphthalene-1-y l) methylene) amino) benzene—sulfonamidesulfonyl) amide has been synthesized. The novel Schiff base H_2_L is used to synthesize novel nano and micro-complexes with CrCl_2_.6H_2_O and CuCl_2_.2H_2_O. The prepared ligand and micro complexes were interpreted by different spectroscopic techniques. The nano-sized Cr and Cu complexes were synthesized in an environmentally friendly manner using *Coriandrum sativum* (CS) media extract in ethanol. The structure, morphologies and particle size of the nano-sized complexes were determined using FT-IR, TEM, and PXRD. The results showed that the nano-domain complexes are on the Sub-nano scale. Furthermore, using TGA, we studied the effect of heat on the size of newly prepared nano-complexes. Experimental data were supported by DFT calculations. The findings revealed that the metal complexes investigated are more stable than the free ligand H_2_L. The antitumor activity was examined before and after heating the nano-complexes at 200 °C. The results reveal the Cr nano complex, after heating, exhibited strong antitumor activity with IC_50_ value (3.349 μg/ml). The tested Cu nano-complex shows good DNA cleavage. The liver cancer and COVID19 proteins were examined using molecular docking to identify the potential binding energy of inhibitors.

## Introduction

Sulfadimidine is a sulfa-based drug that is used to prevent and treat bacterial infections such as influenza, eye infections, actinomices, meningitis infections, and urinary tract infections^1,2^ .They can also be utilized as model molecules to study drug action mechanisms^3^. In addition to other sulfonamides, it has been altered by Schiff-base formation^4^ and the formed complexes with transition elements^5,6^. Because they have multiple potential donor sites, such as amino, pyrimido, and sulfonamidic nitrogen, as well as sulfonyl oxygen atoms, they are good complexing agents. Several Schiff bases generated from sulfa drugs have been produced and employed as ligands in the production of powerful metal complexes^5,7,8^. Schiff base compounds have received a lot of attention from researchers. Schiff bases are useful in many different areas such as polymer stabilizers, catalysts, intermediates in organic synthesis, dyes and pigments^9^. Furthermore, Schiff bases are an essential class of ligands in coordination chemistry. Schiff bases have also been demonstrated to have antibacterial, antifungal, antimalarial, antiviral, anti-inflammatory, and antipyretic effects, among other biological actions^10–15^. The creation of novel compounds with unique physical, chemical, and biological properties results from the synthesis of nano-sized complexes^16^. Plant extracts are used to prepare green nano-domain complexes, which is an environmentally friendly method^17,18^. There is no published literature for Schiff base generated from sulfadimidine sodium with 2-hydroxy-1-naphthaldehyde, its metals, or nano-metal complexes.

Quantum chemical calculations are being used to elucidate molecular interaction mechanisms. Recently, DFT (density functional theory) calculations were performed to comprehend the molecular geometry and molecular electrostatic potential of the investigated compounds^19–23^. Moreover, molecular docking studies were carried out in order to further evaluate and identify the actual molecular recognition interaction between the prepared compounds under study and the predicted target^24–30^.

Therefore, in this study, we focus on the synthesis of a novel Schiff base ligand generated from sulfadimidine sodium and 2-hydroxy-1-naphthaldehyde in addition to the preparation of novel micro and nano-sized complexes. Different physicochemical and spectral analysis techniques were used to characterize the H_2_L ligand and its micro and nano-sized complexes. In addition, molecular modeling was studied to acquire more information about the properties of the prepared compounds under study. Furthermore, the antitumor activities and DNA cleavage of the nano-complexes were evaluated to examine the bio-efficiency. Also, molecular docking studies were used to predicate possible biological applications, using computer software.

## Experimental

### Materials

All chemicals used were obtained from Aldrich or BDH. They included 2-Hydroxy-1-naphthaldehyde, sulfadimidine sodium, CrCl_2_.6H_2_O or CuCl_2_.2H_2_O and LiOH. The solvents, such as absolute ethyl alcohol and Dimethylformamide (DMF) were used as received without pretreatment.

### Instrumentation and spectral measurements

The CHNS contents were evaluated using a Perkin-Elmer elemental analysis (240c). Melting points of all synthesized compounds were measured on Gallenkamp apparatus. ^1^HNMR spectra in DMSO-d^6^ were obtained on a BRUKER 400 MHz. With an (MS-5988 GC–MS) at 70 eV, mass spectra were recorded. The molar conductance was measured on a Jenway conductometer. A Jasco P-530 spectrophotometer was used to acquire electronic spectra. FTIR spectra were recorded on Shimadzu spectrophotometer applying KBr pellet in the range of (4000–400 cm^−1^). With an (MS-5988 GC–MS) at 70 eV, mass spectra were recorded. PXRD patterns were created on a Bruker AXS D8 advanced diffractometer. A Jeol Jem-1200 EX II electron microscope was used to produce the SEM images, with an acceleration voltage of 25 kV. The TEM micrographs were executed using JEOL JEM-1200 EX II, Japan at 60–70 kV. Thermogravimetric analyses of the micro and nanocomplexes were performed in an inert atmosphere on a Shimadzu DTG-60H at 10 °C/min. A Jasco P-530 spectrophotometer was used to acquire electronic spectra. Using Varian E-109 X-band or Q-band spectrophotometers, ESR measurements were done at 77 and 300 K. DPPH was employed as a standard material.

### Synthesis of H_2_L Schiff base ligand

In a round-bottomed flask, a mixture of sulfadimidine sodium (2.7833 g; 10 mmol) and 2-hydroxy-1-naphthaldehyde (1.7218 g, 10 mmol) in 20 mL EtOH was refluxed for 4 h. The reaction mixture was then acidified with 5% HCl to form a neutral (pH=7) solution, and a solid of H_2_L Schiff-base was isolated and recrystallized from ethanol.

### Synthesis of Schiff base complexes

**Figure 1:**
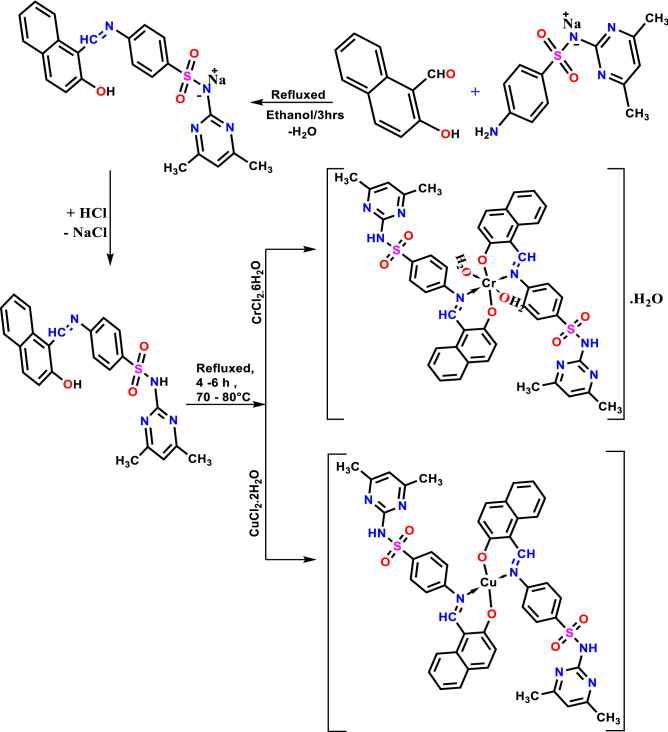
Synthesis of H_2_L and its Cr(II) and Cu(II) complexes.

The H_2_L solution in ethanol was mixed with a solution of CrCl_2_.6H_2_O or CuCl_2_.2H_2_O in ethanol in a 1:1 ratio. The resulting solution was refluxed for 4–6 h at 70 °C. The precipitate was separated by filtration and washed with ethanol, bi-distilled water followed by diethyl ether and lastly dried at room temperature in a desiccator under vacuum (Fig. 1).

### Synthesis of Schiff base nano-complexes

Nano-sized complexes of Cr(II) and Cu(II) were produced in a 1:1 ratio, in ethanol by adding *Coriandrum sativum* (CS)/EtOH media, as previously reported in our publications^31^.

### Computational calculations

The H_2_L and its complexes were optimized using the Gaussian 09 program^32^, using the DFT method comprising the B3LYP/6-311G + (dp) level of theory for all atoms except metal ions and at the B3LYP/LANL2DZ level of theory for metal ions^33^.

### Antitumor activity

The antitumor activity of the ligand and its nanocomplexes was tested against HepG2 cells using cis-platin (cis-diamminedichloroplatinium). The cell lines were obtained from American Type Culture Collection (ATCC; Rockville, MD, USA). The experiments were carried out in the tissue culture unit at Regional Center for Mycology and Biotechnology, Al-Azhar University, Cairo, Egypt. Graphed prism version (San Diego, CA.USA) was used to calculate the IC_50_ values.

### DNA cleavage studies

The DNA cleavage experiment was was carried out using CT-DNA (Calf Thymus DNA) by gel electrophoresis with the corresponding nanocomplex. Test samples were dissolved in DMSO and added separately to the CT-DNA. The sample mixtures were incubated at 37ºC for 2 h. The samples were electrophoresed for 2 h at 100 V on 0.25 g agarose gel using 25 ml of TAE buffer (pH8.0). After electrophoresis, the gel was stained using 10 μg/ml ETBR solution for 10–15 min, then the bands were observed and photographed under UV transilluminator.

### Docking simulation

Molecular docking simulation was executed to anticipate the binding sites and affinity score, using MOE2019 version software^34^, and the crystal structures of different targets were reclaimed from the protein data bank (https://www.rcsb.org) in the pdb format. The target proteins include human methionine adenosyl-transferases (PDB ID: 5A19) which is tightly linked to liver cancer^35^, and the crystal structure of COVID-19 main protease viral protein (PDB ID: 6lu7)^36^.

## Results and discussion

### Analytical data

The CHNS contents, chemical formula, melting points, Color and yields of the synthesized compounds are postulated in Table 1. This illustrates the purity of the ligand and its complexes.

**Table 1:** Elemental analysis and physical properties of the study compounds.

Compound	M.F	M.Wt. (g/mol)	Color	m.p. (^o^C)	Yield (%)	Found value % (theoretical value) %				Ω_m_ (ohm^−1^ cm^2^mol^−1^)
						% C	% H	% N	% S	
H_2_L	C_23_H_20_N_4_O_3_S	432.50	Yellow	185	67	63.69 (63.87)	4.48 (4.66)	12.73 (12.95)	7.52 (7.41)	0.7
[CrL_2_(H_2_O)_2_].H_2_O	C_46_H_44_CrN_8_O_9_S_2_	969.02	Dark brown	> 300	73	57.21 (57.02)	4.51 (4.58)	11.74 (11.56)	6.59 (6.62)	4.58
CuL_2_	C_46_H_38_CuN_8_O_6_S_2_	926.53	Red	> 300	76	59.83 (59.63)	4.08 (4.13)	12.23 (12.09)	6.87 (6.92)	5.35

### ^1^H-NMR interpretations

The ^1^H NMR spectra of the synthesized H_2_L ligand were detected in DMSO and D_2_O (Figs. S1 and S2). For H_2_L, The CH_3_ and OH protons signals were detected as a singlet at δ2.21 and 9.64 ppm, respectively. The imine (–CH=N) signal was observed at δ 8.58 ppm^37^. The NH proton signal was observed at δ 10.61 ppm^38^. The aromatic proton signals of the H_2_L ligand were detected between δ 7.24 and 7.91 ppm^39^. The signal at δ 2.51 ppm for DMSO-d^6^ ^37^. The hydroxyl group signal has completely disappeared upon the addition of D_2_O, indicating the purity of the prepared ligand.

### Molar conductivity analysis

The molar conductance (Ω_m_) of the prepared ligand H_2_L and its micro-complexes in 10^–3^ M DMSO solution lie in the 0.7 to 5.35 Ohm^−1^ cm^2^ mol^−1^ range (Table 1), revealing the non-electrolytic nature of these compounds^40^.

### IR spectra

#### Infrared spectral studies of micro-complexes

**Figure 2:**
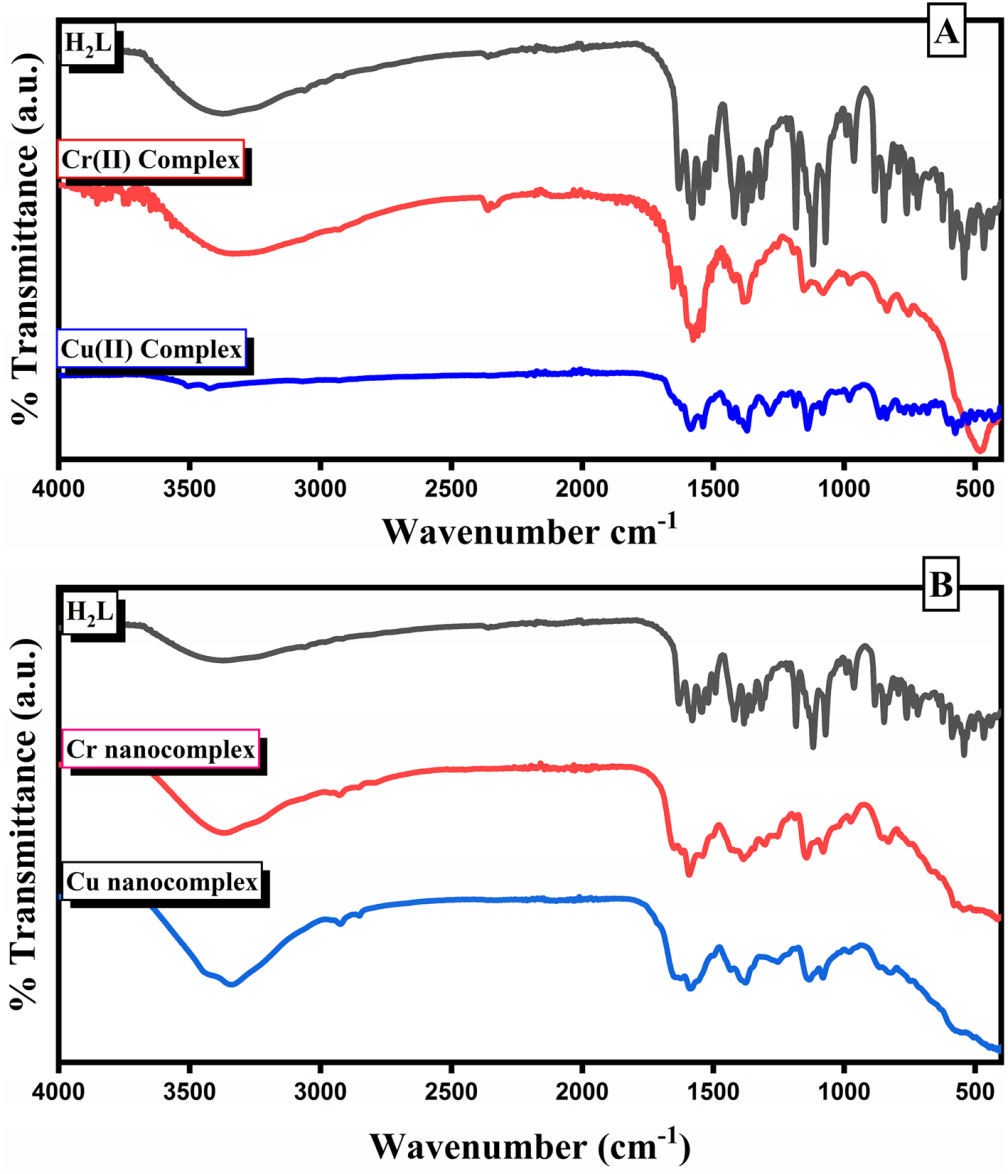
FT-IR spectra of H_2_L and its micro-complexes (**A**) and nano-complexes in CS media (**B**).

The infrared spectra of the investigated ligand and its metal(II) Chelates are depicted in (Fig. 2A). The H_2_L ligand spectral exhibited absorption bands at 3200, 1384, and 1156 cm^−1^ that is, respectively, assigned to υ(OH), υ_as_(SO_2_) and υ_sy_(SO_2_) groups. The synthesis of the H_2_L ligand is revealed by the imine signal of the ligand (C=N) at 1633 cm^−1^; however, in the Cr(II) and Cu(II) complexes, this signal shifted to 1607 and 1613 cm^−1^, suggesting that imine nitrogen is involved in coordination^41^. The phenolic (O–H) of the H_2_L disappeared in the Cr(II) and Cu(II) complexes, showing proton replacement during complex formation. The three characteristic peaks of υ(NH) at 3457, υ_as_(SO_2_) at 1384 cm^−1^ and υ_sy_(SO_2_) at 1156 cm^−1^ in the spectra of the Cr(II) and Cu(II) micro-complexes and their free ligand all appear at the same position, excludes the coordination through the NH and SO_2_ groups^42^. The new bands in the IR spectra of the prepared compounds at 520–528 and 570–573 cm^−1^ are ascribed to (M–N) and (M–O), respectively. Furthermore, in the spectra of the Cr(II) complex, the broadband near 3477 could be attributed to the υ(OH_2_) (Table 2).

**Table 2:** Essential vibrational frequencies (cm^−1^) of H_2_L and its micro-complexes.

No	Compound	υ(C=N)	υ(O–H)	υ_as_(SO_2_)	υ_sy_(SO_2_)	υ(H_2_O)	υ(M–O)	υ(M–N)
IR bands (cm^−1^)								
I	H_2_L	1633	3200	1384	1156	–	–	–
II	CrL_2_	1607	–	1384	1152	3477	573	528
III	CuL_2_	1613	–	1384	1154	–	570	520

#### Infrared spectral studies of nano-complexes

The FTIR spectra of the Cr and Cu nano-domain complexes generated in CS/EtOH media are given in (Fig. 2B), which reveals the binding modes of the Cr and Cu nano-domain complexes, which are corroborated by a change in band position of the nano-domain complexes when compared to the free ligand.

### Mass spectral data

**Figure 3:**
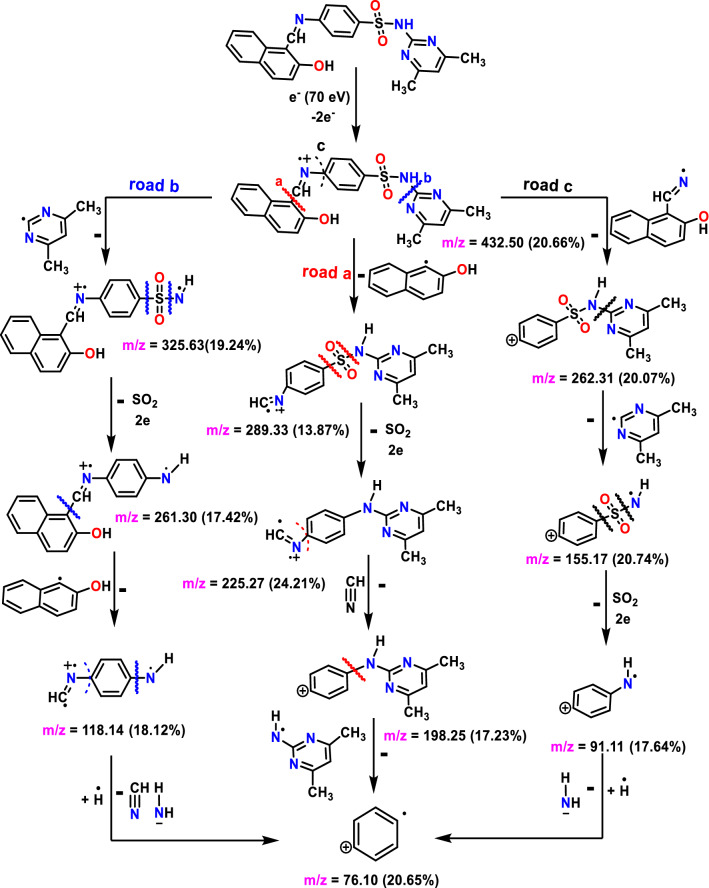
Suggested MS fragmentation pattern of Schiff base ligand.

MS of the synthesized H_2_L and its Cr and Cu complexes are shown in (Fig. S3). The MS of H_2_L exhibited a molecular ion peak (m/z)=432.45, which is in good agreement with the proposed molecular weight (Fig. S3A). The proposed fragmentation pattern of the H_2_L ligand was given in (Fig. 3). The mass spectrum of the Cr(II) and Cu(II) complexes displayed molecular ion peaks at m/z 968.27 and 518.92, respectively, approving the molecular weight and the existence of Cr and Cu isotope peaks at m/z 54 and 65, respectively (Fig. S3B, C).

### X‑ray study

**Figure 4:**
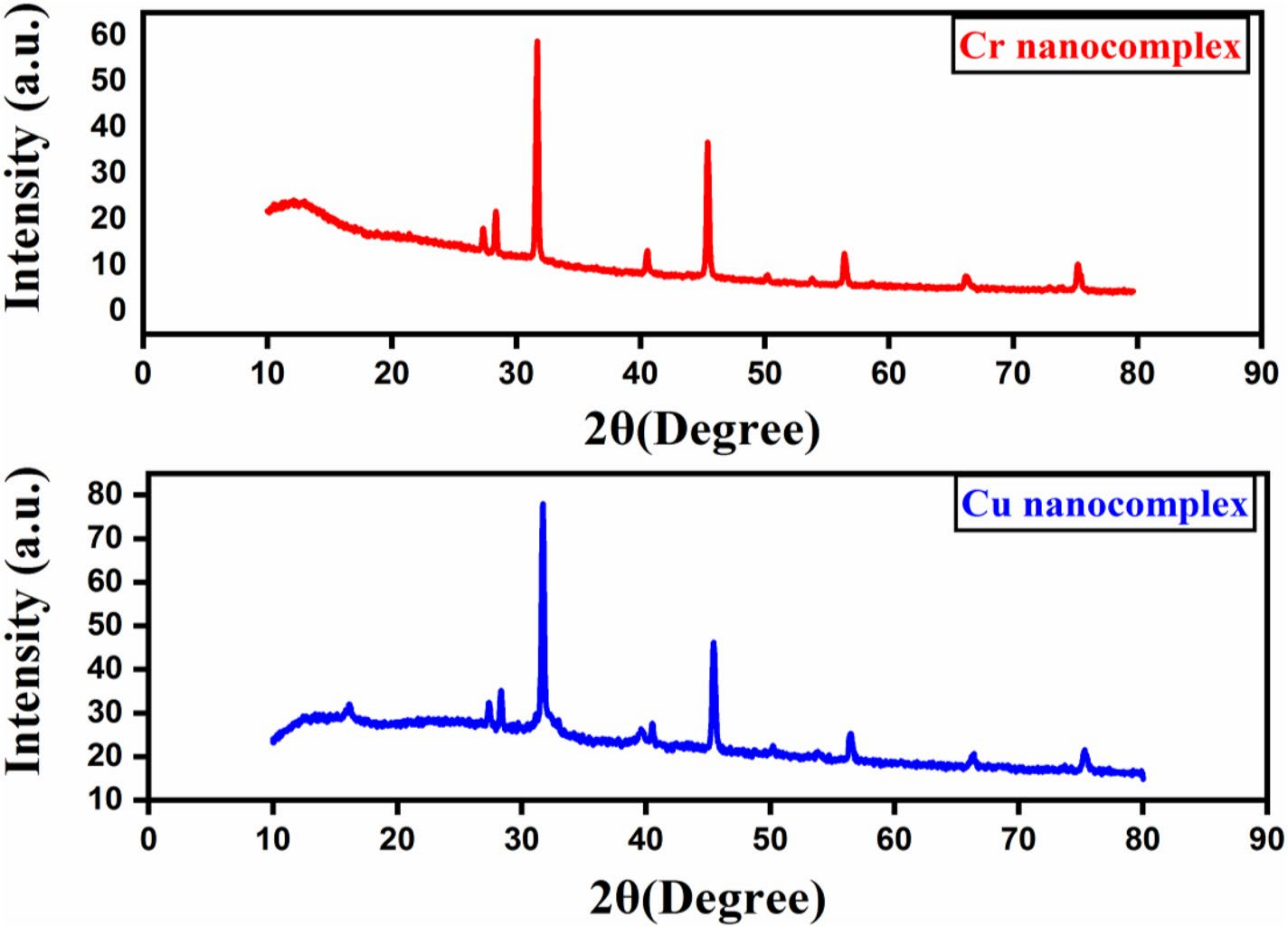
XRD patterns of Cr and Cu nano-complexes in CS media.

The X-ray diffraction pattern is an analytical technique that was used to learn more about the chemistry and crystallographic structure of the obtained Cr and Cu nano-complexes (Fig. 4). The crystalline phase of the prepared Cr and Cu nano-complexes in CS/EtOH media was revealed by their sharp and high-intensity diffraction peaks. The nano crystallite sizes of the Cr and Cu nano-complexes were calculated using the Scherrer formula^17,37,43^, which are 42.5 and 39.2 nm, respectively, which lay inside the range of the nano-scale.

### SEM analysis of micro-complexes

**Figure 5:**
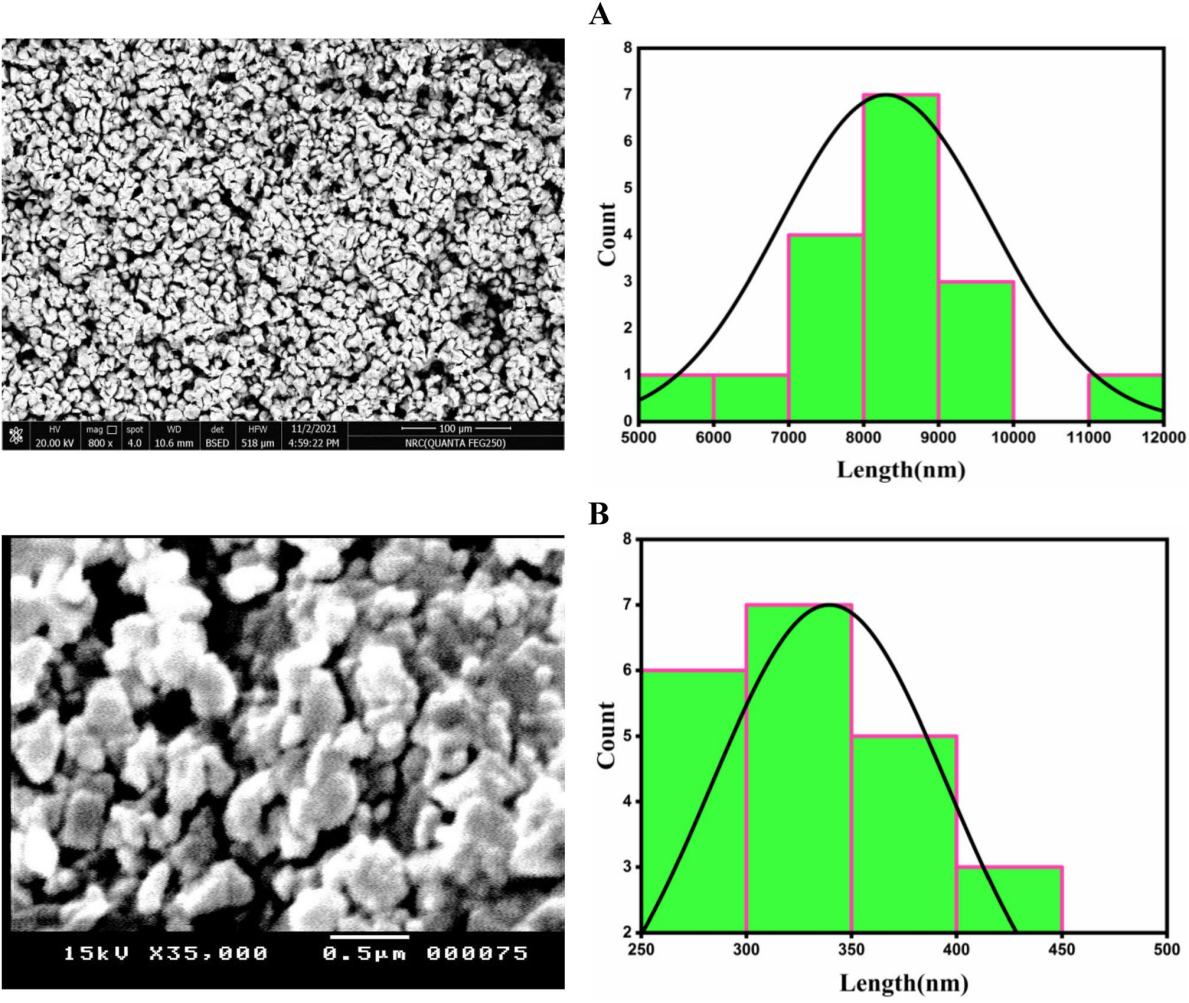
SEM micrographs and particle size distribution curves for Cr(II) complex (**A**) and Cu(II) complex (**B**).

The morphologies and particle size of the Cr(II) and Cu(II) micro-complexes have been demonstrated by the scanning electron microscope (SEM). Figure 5 depicts the SEM photographs of the synthesized Cr(II) and Cu(II) Schiff base complexes. In the pictograph, we observed that the produced complexes are arranged in a regular matrix. This suggests that Cr(II) and Cu(II) complexes have homogeneous phase material. The Cr(II) complex has a seashell-like shape with an average particle size of 8 µm. The images of the Cu (II) complex display a spherical shape with an average particle size of 0.33 µm. This indicates that Cr(II) and Cu(II) complexes are found in the microdomain.

### TEM measurement of nano-complexes

**Figure 6:**
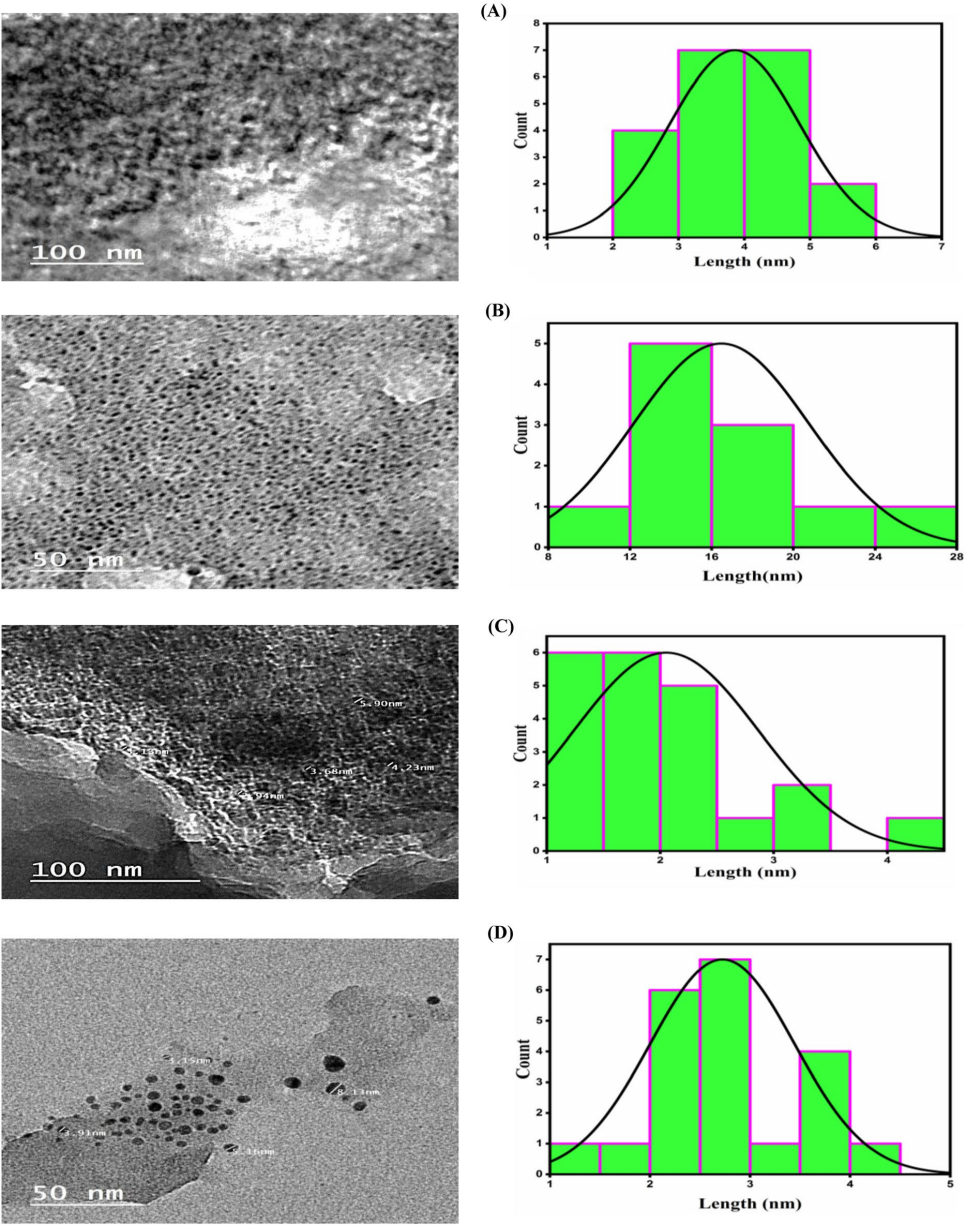
TEM pictures and particle size distribution curves for Cr nano-complex (**A** & **C**) and Cu nano-complex (**B** & **D**) in *CS* media before and after heating, respectively.

The transmission electron microscopy (TEM) technique was used to examine the morphologies and particle size of the Cr and Cu nano-sized complexes produced in *Coriandrum sativum* (CS) media before and after heating the nano-sized chelates at 200 °C for 2 h. The images of the Cr and Cu nano-complexes show that these complexes have a spherical shape. Moreover, the calculated histogram for the average particle size of the prepared nano-sized chelates before heating was 3.85 and 16.42 nm (Sub-nano) for Cr and Cu nano-sized chelates, respectively. While heating Cr and Cu nano-complexes to 200 °C resulted in particle sizes of 2.05 nm and 2.72 nm (Sub-nano), respectively, it also indicates that the particle size of Cr and Cu nano-sized chelates decreases with increasing temperature (Fig. 6).

### Thermal analysis

#### Thermal analysis of micro-complexes

The results of thermogravimetric analysis of mononuclear Cr(II) and Cu(II) complexes are collected in (Table 3). The thermogram of Cr(II) complex comprises four decomposition steps. The weight losses in the temperature range of 45–317 °C are attributed to the removal of one lattice H_2_O molecule, two coordinated H_2_O molecules and 2C_6_H_8_N_2_. In the range of 317–395 °C, the loss in weight corresponds to the loss of 2SO_2_ and 2C_6_H_6_. Another loss of 2C_10_H_8_ molecules occurs at temperatures ranging from 395 to 578 °C. At higher temperatures (578–736 °C), the found weight loss associated with this step may be attributed to the loss of the 2HCN molecules. The thermal decomposition of the Cu(II) complex has three main degradation steps. The Cu(II) complex exhibits thermal stability up to 400 °C, indicating that crystalline water molecules and coordinated water molecules do not exist in this complex. The first stage of decomposition occurs at the temperature range of 41–451 °C, which corresponds to the elimination of 2C_6_H_8_N_2_. The removal of 2C_6_H_6_, SO_2_ and HCN take place within the temperature range 451–702 °C followed by the loss of 2C_11_H_7_NO within the temperature range 702–860 °C. In Cr(II) and Cu(II) complexes, the remaining residues are metal oxides. These results are following the composition of the complexes. A representative TG/DTA diagram is given in (Fig. S4A, B).

**Table 3:** Thermogravimetric results of metal complexes of the ligand H_2_L.

Complexes	Steps	DTG Endo↓, Exo↑	Temp. range (°C)	Weight loss (%)		Assignment
				Found	Calc	
[CrL_2_(H_2_O)_2_].H_2_O	1st	272**↓**	45–317	28.39	27.68	3H_2_O + 2C_6_H_8_N_2_
	2nd	358**↓**	317–395	29.95	29.34	2SO_2_ + 2C_6_H_6_
	3rd	572**↓**	395–578	25.52	26.03	2C_10_H_8_
	4th	712**↓**	578–736	6.25	5.57	2HCN
	Residue		736–1000	15.56	15.68	Cr_2_O_3_
CuL_2_	1st	428**↓**	41–451	22.85	23.10	2C_6_H_8_N_2_
	2nd	640**↓**	451–702	26.44	26.68	2C_6_H_6_ + SO_2_ + HCN
	3rd	766**↓**	702–860	37.98	36.51	2C_11_H_7_NO
	Residue		860–1000	8.32	8.58	CuO

#### Thermal analysis of nano-complexes

The current thermal analysis techniques aim to acquire more about the chemistry of the Cr and Cu nano-complexes generated in CS/EtOH media by measuring the size of the nano-complexes at each step of thermal heating to determine how the temperature influences the size of the nano-complexes. The particle sizes of Cr and Cu nano-complexes were calculated before and after heating at 200 °C (Tables 4 and 5). After heating at 200 °C, the thermograms of Cr and Cu nano-complexes revealed a reduction in the particle size at each of the thermal heating steps. The heat treatment reduces the accumulation of nano-complexes and thus reduces particles size. Cr and Cu nano-complexes are present in sub-nano structures (Fig. S4C, F).

**Table 4:** TGA steps and the particle size of Cr and Cu nanodomain of the ligand H_2_L before heating.

Nano complex	Media	Temperature range (^o^C)	Loss in wt. % (Found %)	Particle Size (nm)
Cr nanocomplex	*Coriandrum sativum (CS)*	29 – 158	16.20	3.22
		158 – 286	9.27	2.92
		286– 638	22.32	2.27
		638 – 1000	26.04	1.68
Cu nanocomplex		38–146	9.95	14.78
		146–304	13.61	12.76
		304–567	19.15	10.31
		567–1000	22.45	7.99

**Table 5:** TGA steps and the particle size of Cr and Cu nanodomain of the ligand H_2_L after heating.

Nano complex	Media	Temperature range (^o^C)	Loss in wt.% (Found %)	Particle size (nm)
Cr nanocomplex	*Coriandrum sativum (CS)*	35 – 156	14.19	1.76
		156 – 324	6.93	1.63
		324– 602	19.97	1.31
		602– 1000	26.59	0.96
Cu nanocomplex		35–199	14.76	2.32
		199–520	27.35	1.68
		520–1000	27.76	1.21

### Electronic spectroscopy

The H_2_L Schiff base ligand shows four absorption bands in DMF^44^. H_2_L Schiff base ligand displayed absorption bands at 386, 445, 468 and 539 nm, which may be assigned to π→π* transition of the benzene rings, n→π* of imine (C=N), n→π* of the phenolic group and charge-transfer (CT), respectively. The Cr(II) complex revealed absorption bands at 334, 404, 434, 485 and 540 nm that can be attributed to π→π* transition of the benzene rings, n→π* of imine (C=N), n→π* of the phenolic group, charge transfer CT (M→L) and ^5^Eg→^5^T_2_g(D) transition, respectively. These absorption bands resemble an octahedral geometry^45^. The spectrum of the Cu(II) complex exhibits three bands at 313, 364 and 412 nm, which can be attributed, respectively, to the π→π* transition of the benzene rings, n→π* of imine and phenolic groups. Moreover, a broad band at 543 nm has been recorded for the ^2^T_2_→^2^E(D) transition. These electronic bands resemble a tetrahedral geometry^46,47^. The electronic spectrum of green synthesized nano-complexes revealed a brand that is associated with the surface plasmon resonance (SPR) ^48^, confirming the reduction process and formation of nano-complexes. The Cr nano-complex exhibits bands at 329, 381, 439 and 523 nm transitions, which are connected to π→π* transitions of the benzene rings, n→π* of imine and phenolic groups and ^5^Eg→^5^T_2_g(D) transitions, respectively. This suggests that the Cr nano-complex is an octahedral structure. The three bands in the spectrum of the Cu nano-complex detected at 364, 405 and 536 nm are attributed to n→π* of imine and phenolic groups and ^2^T_2_→^2^E(D) transitions, respectively, and suggest a tetrahedral geometry (Fig. S5) & (Table 6).

**Table 6:** UV–Visible spectra of H_2_L, its micro and nano-complexes.

Compound	Spectral bands (nm)	Wavenumber (cm^−1^)	ε_max_ (L mol^−1^ cm^−1^)	Assignment	Proposed geometry
H_2_L	386	25,906	12,610	π → π*	–
	445	22,471	9940	n → π*	
	468	21,367	9270	n → π*	
	539	18,552	1169	Charge transfer (CT)	
[CrL_2_(H_2_O)_2_].H_2_O	334	29,940	7280	π → π*	Octahedral
	404	24,752	3540	n → π*	
	434	23,041	3030	n → π*	
	485	20,618	1730	CT (M → L)	
	540	18,518	1183	^5^Eg→^5^T_2_g(D)	
CuL_2_	313	31,948	11,890	π → π*	Tetrahedral
	364	27,472	5630	n → π*	
	412	24,271	3080	n → π*	
	545	18,348	1104	^2^T_2_→^2^E(D)	
Cr nanocomplex	329	30,395	9630	π → π*	Octahedral
	381	26,246	6470	n → π*	
	439	22,779	3918	n → π*	
	523	19,120	1604	^5^Eg→^5^T_2_g(D)	
Cu nanocomplex	364	27,472	7843	n → π*	Tetrahedral
	405	24,691	5860	n → π*	
	536	18,656	1303	^2^T_2_→^2^E(D)	

### EPR spectroscopy

**Figure 7:**
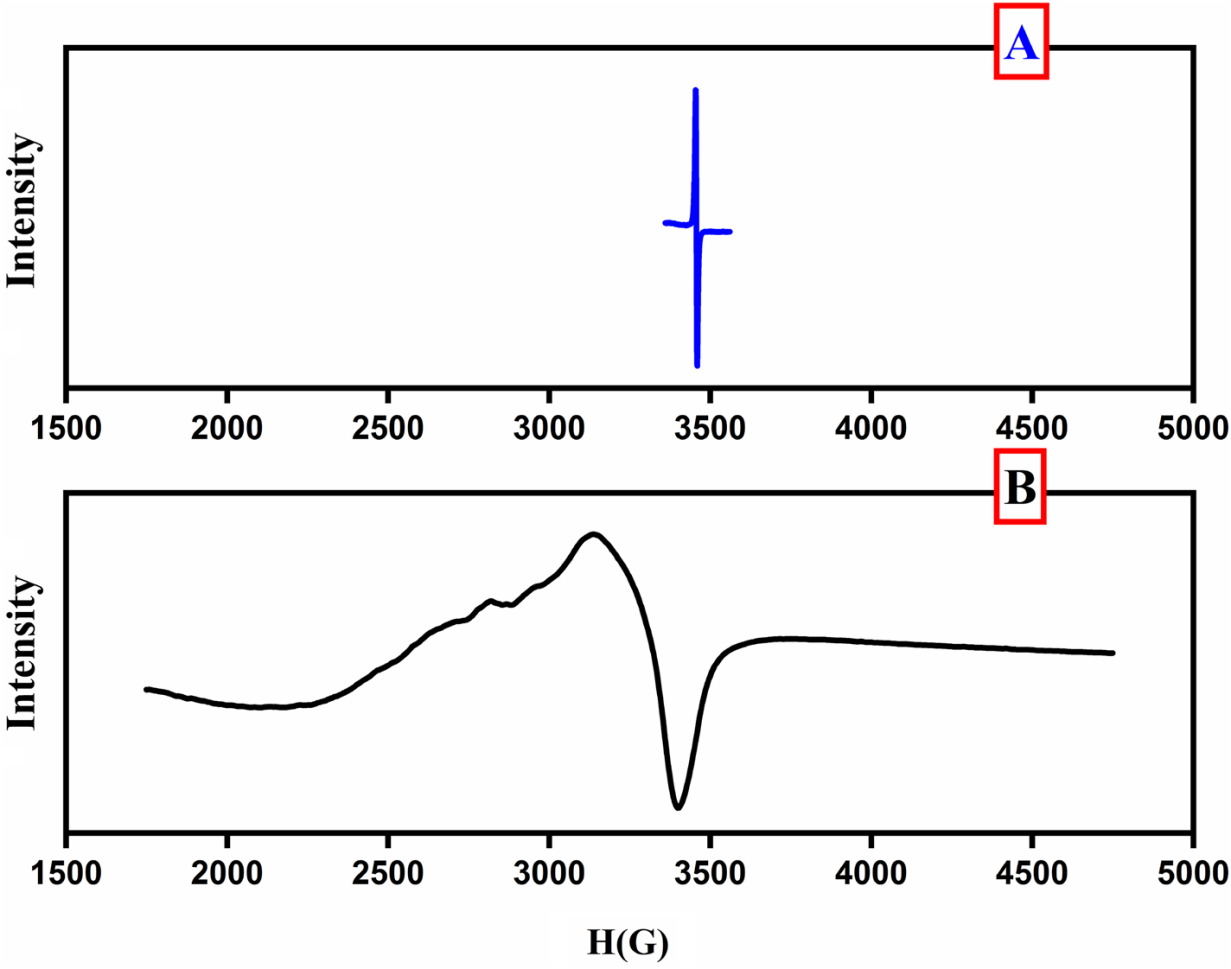
EPR spectra of the standard (DPPH) (**A**) and CuL_2_ (**B**).

The X-band EPR spectra of Cu(II) complex were recorded at room temperature (Fig. 7). The effective g_eff_-value of the observed EPR spectrum of the prepared Cu(II) complex was determined and listed in (Table 7). The investigated Cu(II) complex is suggested to have tetrahedral geometry based on the shape of the observed EPR signals and g_eff_-value. According to the results obtained, the g║ value for the Cu(II) complex is lower than 2.3, revealing that the metal–ligand bonds are covalent. The geometric parameter G factor defined as G=(g_||_–2.0023)/(g_⊥_–2.0023), which is 0.355, suggests that the exchange interaction is detected in the Cu(II) complex and the exchange interaction was operative between copper centers in the present Cu(II) complex^49,50^.

**Table 7:** EPR parameters for Cu(II) complex.

Complex	g║	g_┴_	g_eff_	G
CuL_2_	2.075	2.211	1.515	0.355

### Molecular modeling

#### *Geometry optimization of the H*_*2*_*L ligand*

**Figure 8:**
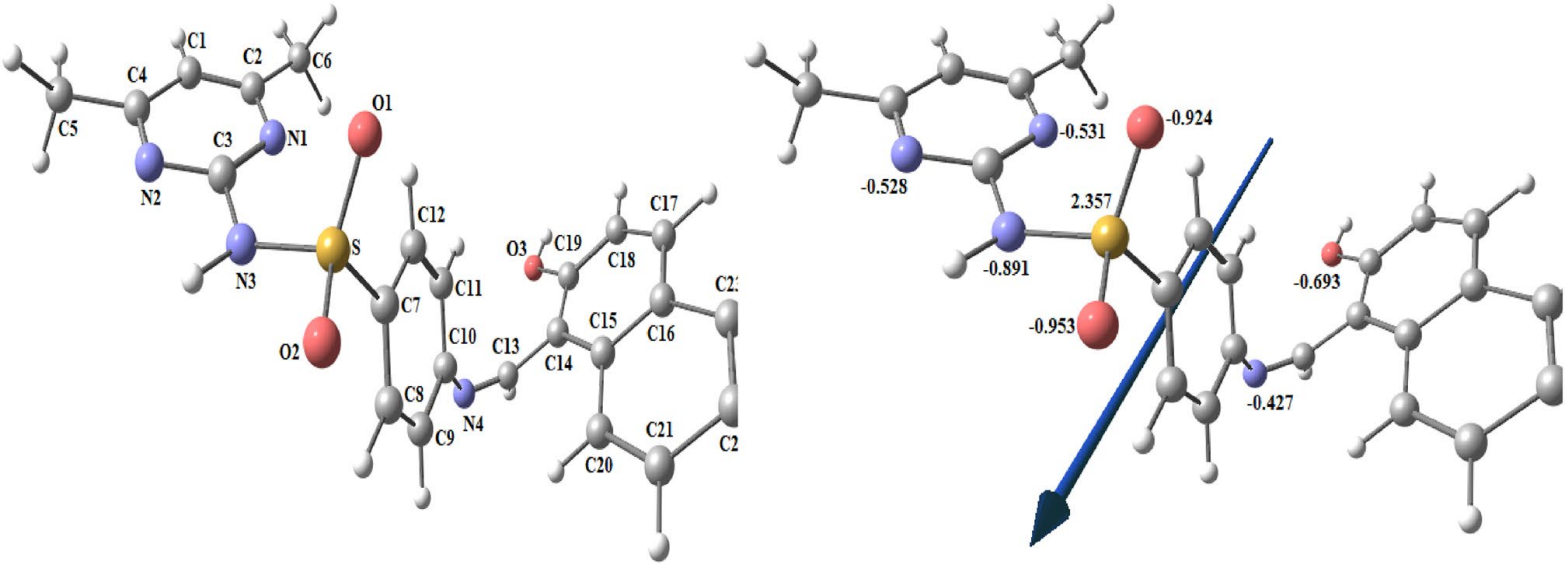
Optimized structures of H_2_L, dipole moment vector, and the charges on active centers of H_2_L.

Figure 8 shows the molecular optimization of the H_2_L by using the B3LYP method and 6–311 + G(d,p) basis sets. The charges calculated from the natural-bond-orbital method (NBO) are: O1(− 0.693), O2(− 0.953), O3(− 0.924), N1(− 0.531), N2(− 0.528), N3(− 0.891), N4(− 0.427) and S(2.357). Metal ions coordinate to O3 and N4 atoms.

#### Geometry optimization of the metal complexes

Complexes were optimized using the Gaussian 09 program, using the DFT method comprising the B3LYP/6-311G + (dp) level of theory for all atoms except metal ions and at the B3LYP/LANL2DZ level of theory for metal ions. The computed theoretical infrared frequencies ensure the absence of imaginary frequencies.

##### Optimization of [CrL_2_(H_2_O)_2_].H_2_O

**Figure 9:**
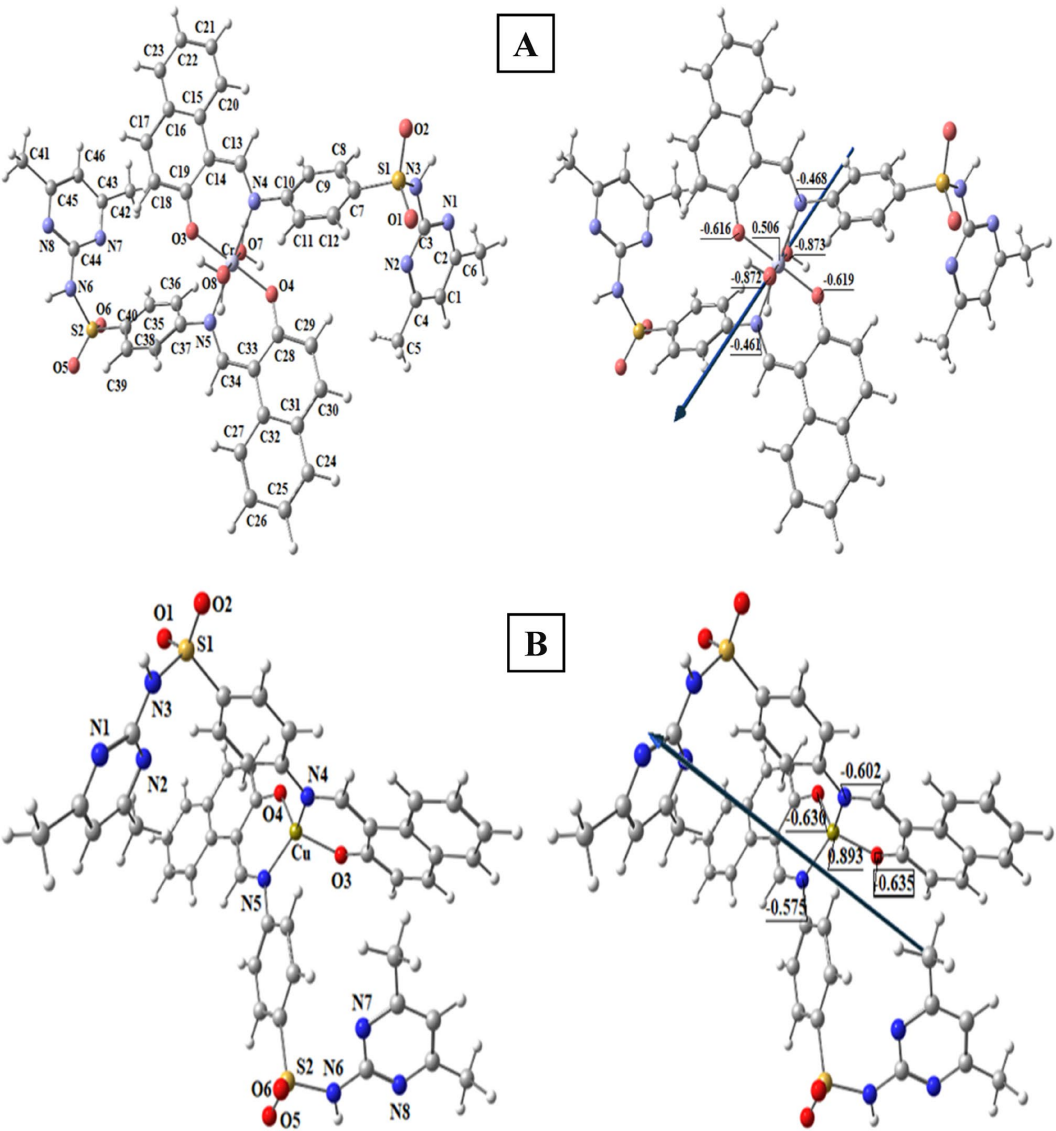
The optimized structures, dipole moment vector, and the charges on active centers of [CrL_2_(H_2_O)_2_].H_2_O (**A**), [CuL_2_] (**B**).

Figure 9A displays the molecular optimization of the complex [CrL_2_(H_2_O)_2_].H_2_O as the lowest-energy configuration. The chromium metal ion is six-coordinated in an octahedral geometry, with atoms O3, N4, O4, and N5 having a dihedral angle of 0.730° (Table 8). The distance between N4 and O1 in the ligand (3.715 Å) is reduced in the [CrL_2_(H_2_O)_2_].H_2_O complex to 2.786 and 2.779 Å for N4–O3 and N5–O4, respectively, as a result of complex formation. The charges calculated from the natural-bond-orbital method (NBO) on the coordinated atoms are Cr (+ 0.506), O3 (− 0.616), O4 (− 0.619), N4 (− 0.468), N5 (− 0.461), O7 (− 0.873) and O8 (− 0.872).

**Table 8:** Bond lengths (Å) and bond angles (^o^) of optimized [CrL_2_(H_2_O)_2_].H_2_O.

Bond	Bond length(Å)	Bond	Bond length (Å)	
	Complex		H_2_L	Complex
Cr–N4	2.072	Cr–N5	–	2.066
Cr–O3	1.939	Cr–O4		1.939
Cr–O7	2.208	N4–O3	3.715	2.786
Cr–O8	2.201	N5–O4	3.715	2.779

Angle	Angle (°) Complex	Angle	Angle (°) Complex	
N4-Cr-O3	87.95	O8–Cr–N4	95.45	
N5-Cr-O4	87.81	O8–Cr–N5	84.61	
N5-Cr-O3	91.80	O8–Cr–O3	85.74	
N4-Cr-O4	92.44	O8–Cr–O4	93.26	
O7-Cr-N4	84.23	N4–Cr–N5	179.7	
O7-Cr-N5	95.72	O3–Cr–O4	179.0	
O7-Cr-O3	95.41	O7–Cr–O8	178.8	
O7-Cr-O4	85.60	N4–O3–N5–O4	0.730*	

*Dihedral angle.

##### Optimization of [CuL_2_]

Figure 9B shows the molecular optimization of the complex [CuL_2_] as the lowest-energy configuration. The copper metal ion is four-coordinated in a tetrahedral geometry (Table 9). The distance between N4 and O1 in the ligand (3.715 Å) is reduced in the [CuL_2_] complex to 2.913 and 2.821 Å for N4–O3 and N5–O4, respectively, as a result of complex formation. The charges computed from the natural-bond-orbital method (NBO) on the coordinated atoms are Cu (+ 0.893), O3(− 0.635), N4(− 0.602), O4(− 0.630) and N5(− 0.575).

**Table 9:** Bond lengths (Å) and bond angles (^o^) of optimized CuL_2_.

Bond	Bond length(Å)	Bond	Bond length(Å)	
	Complex		H_2_L	Complex
Cu–O3	1.978	N4–O3	3.715	2.913
Cu–O4	1.849	N5–O4	3.715	2.821
Cu–N4	1.955			
Cu–N5	1.881			

Angle	Angle (°) Complex	Angle	Angle (°) Complex	
N4–Cu–O3	95.58	O3–Cu–N5	110.4	
N4–Cu–N5	127.2	O3–Cu–O4	115.31	
N4–Cu–O4	111.1	O4–Cu–N5	98.27	

#### HOMO/LUMO energy evaluation 

**Figure 10:**
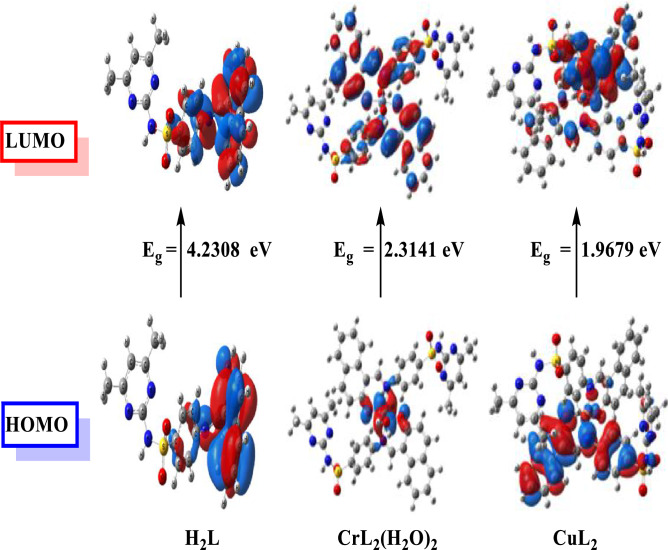
MO and their energies for, (H_2_L), complexes [CrL_2_(H_2_O)_2_].H_2_O and [CuL_2_]).

An important method for analyzing the chemical behavior of ligands and complexes is HOMO/LUMO energy evaluation (Fig. 10). The ability of the compound to acquire electrons is expressed by the LUMO energy, whereas its ability to donate electrons is expressed by the HOMO energy. The total energies of the Cr(II) and Cu(II) complexes are lower than those of the free ligand, indicating that they are more stable. In addition to the frontier molecular orbitals are used in estimation of other chemical parameters such as dipole moment, energy gap (E_g_), ionization potential (I), electronegativity (χ), Chemical hardness (η), Chemical softness (S), electrophilicity (ω), electron affinity (A), chemical potential (μ) are tabulated in (Table 10). It was reported that a molecule with a high energy gap (E_g_) value resulted in a hard molecule with low reactivity. CuL_2_ complex has a higher softness (S) value, suggesting a higher chemical reactivity.

**Table 10:** Calculated electronic parameters of the ligand (H_2_L), complexes [CrL_2_(H_2_O)_2_].H_2_O and [CuL_2_]).

Property	H_2_L	[CrL_2_(H_2_O)_2_].H_2_O	[CuL_2_]
The total energy E (a.u.)	− 1731.338	− 3700.702	− 3657.610
HOMO (eV)	− 5.8499	− 4.3128	− 5.6720
LUMO (eV)	− 1.6191	− 1.9987	− 3.7041
E_g_=E_LUMO_−E_HOMO_ (eV)	4.2308	2.3141	1.9679
Dipole moment (Debye)	6.1304	3.5085	4.8415
Ionization potential I=− E_HOMO_	5.8499	4.3128	5.672
Electron affinity A=-E_LUMO_	1.6191	1.9987	3.7041
Electronegativity χ=(I + A)/2	3.7345	3.1558	4.6881
Chemical hardness η=(I−A)/2	2.1154	1.1571	0.9840
Chemical softness S=1/2η	0.2364	0.4321	0.5082
Chemical potential μ=− χ	− 3.7345	− 3.1558	− 4.6881
Electrophilicity ω=μ^2^/2η	3.2964	4.3035	11.1682

#### Electrostatic potential map (MEP) 

**Figure 11:**
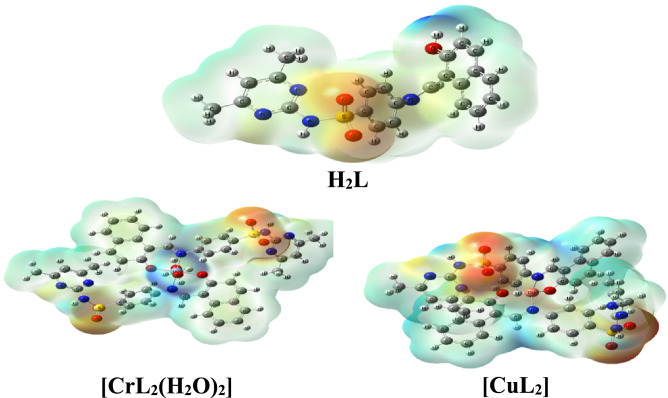
Molecular electrostatic potential (MEP) surface for H_2_Land complexes [CrL_2_(H_2_O)_2_].H_2_O and [CuL_2_]).

The electrostatic potential mapping is based on the total electron density surface, which displays electrostatic potential distribution, size, structural shape and dipole moments. Figure 11 displays the MEP for the investigated ligand and its complexes. Red, blue, yellow, and green color zones on the MEP surface represent, respectively, electron-rich, electron-poor, moderately electron-poor, and neutral zones. Positive potential zones are found over hydrogen atoms in the MEP, while negative potential regions are found over electronegative atoms (oxygen and nitrogen). The green region predominated in the MEP surfaces, corresponding to a potential halfway between the two extremes of red and dark blue color.

### Antitumor activity

**Figure 12:**
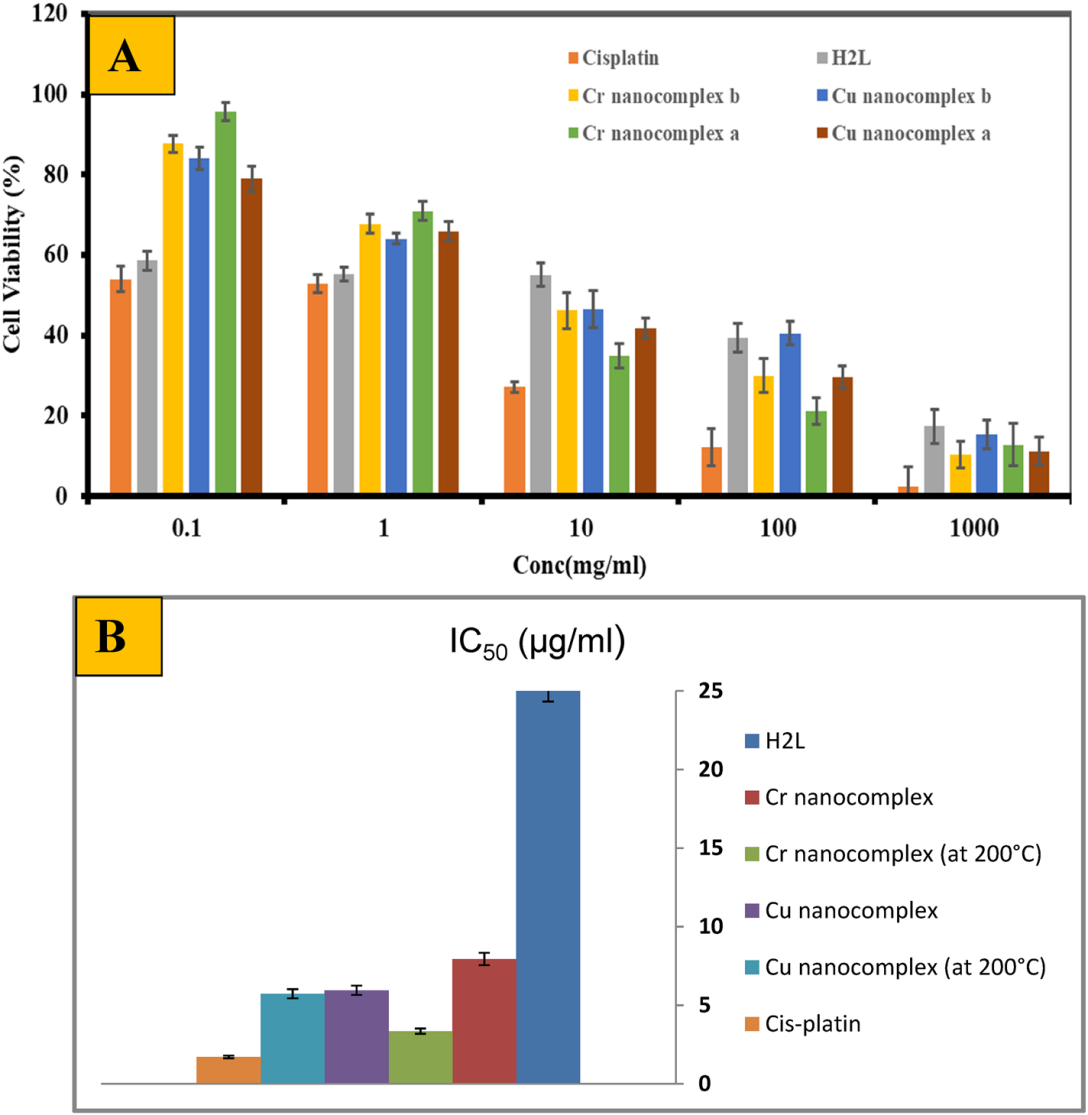
The cell viability of H_2_L and its nano-complexes prepared in CS/EtOH media versus cisplatin drug (**A**). IC_50_ values of H_2_L and its nano-complexes (before and after heating) on HepG-2 cell line (**B**).

The ligand H_2_L and its Cr and Cu nano-complexes prepared in CS/EtOH media before and after heating the nanodomain complexes at 200 °C were measured against a cell line (HepG-2) to establish in vitro antitumor efficiency (Fig. 12A) and compared with the cis-platin anticancer drug (IC_50_ ~ 1.714 μg/ml). The antitumor and growth-inhibitory effects were investigated by using the IC_50_ approach, which estimates the concentration of a drug that reduces cell lineout growth by 50%. Compounds with IC_50_ values less than 5.00 μg/ml, within 5.00–10.00 μg/ml and between 10.00 and 25.00 μg/ml are considered strong, moderate, and mild antitumor activity, respectively^51^. The nano-sized Cr complex, after heating at 200 °C, exhibited strong antitumor activity with IC_50_ value (3.349 μg/ml). The nano-sized Cr(II) complex (before heating) and Cu(II) complex (before and after heating) displayed moderate antitumor activity, with IC_50_ values ranging from 5.73 to 7.49 μg/ml, whereas the H_2_L ligand showed weak antitumor activity, with IC_50_ value of 25.6 μg/ml (Fig. 12B). The results reveal that the nanodomain complexes generated in CS/EtOH media after heating at 200 °C have greater efficiency compared to the nano-complexes without heating. This may be due to the heating nano-complexes having a higher ability to bind DNA than others examined due to their small particle size, which can also be used extensively in economic anticancer studies by specialists.

### DNA cleavage studies

**Figure 13:**
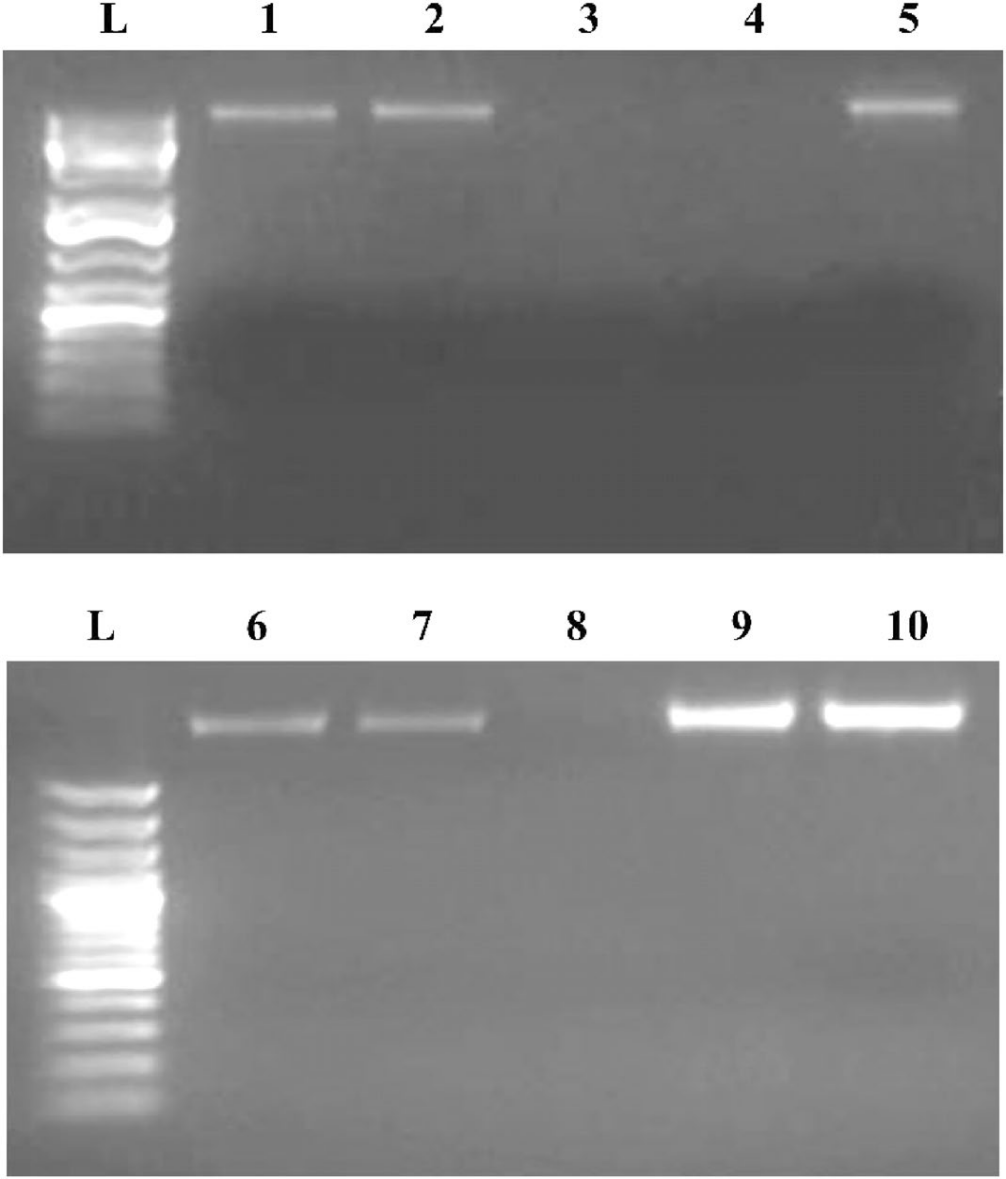
Gel electrophoresis diagram illustrating the cleavage of DNA by Cu nano-complex after incubation at 37 °C for 1 h. Lane L- marker 1 kb DNA Ladder, lane 1 DNA control, lane 2 DNA + DMSO, lane 3: 400 ng DNA + 2 mg/ml of nano-complex, lane 4: 400 ng DNA + 1 mg/ml of nano-complex, lane 5: 400 ng DNA + 0.5 mg/ml of nano-complex, lane 6: DNA control, lane 7: DNA + DMSO, lane 8: 200 ng DNA + 1 mg/ml of nano-complex, lane 9: 400 ng DNA + 1 mg/ml of nano-complex, lane 10: 800 ng DNA + 1 mg/ml of nano-complex.

DNA interacts with nano-domain complexes in many different ways, and these interactions significantly affect the structure and function of DNA^52^. The Cu nano-domain complex was investigated for its DNA cleavage activity using agarose gel electrophoresis, which was used with different and constant concentrations of the copper nano-domain complex with constant and different concentrations of DNA, respectively, to determine whether the nano-domain Cu complex prepared in CS/EtOH media has any impact on DNA. According to the results, the Cu nano-domain complex could cleave DNA at high concentrations (2 and 1 mg/ml) in the presence of a constant concentration of DNA and variable concentrations of the Cu nano-domain complex (Fig. 13 lane 3,4); Furthermore, when a fixed concentration of Cu nano-domain complex is present in the presence of varying amounts of DNA, the Cu nano-domain complex can cleave DNA at low concentrations of DNA (Fig. 13 lane 8]; under the experimental conditions and full length images (Figs. S6 and S7).

### Molecular docking simulation with liver cancer and COVID-19 protein receptors

**Figure 14:**
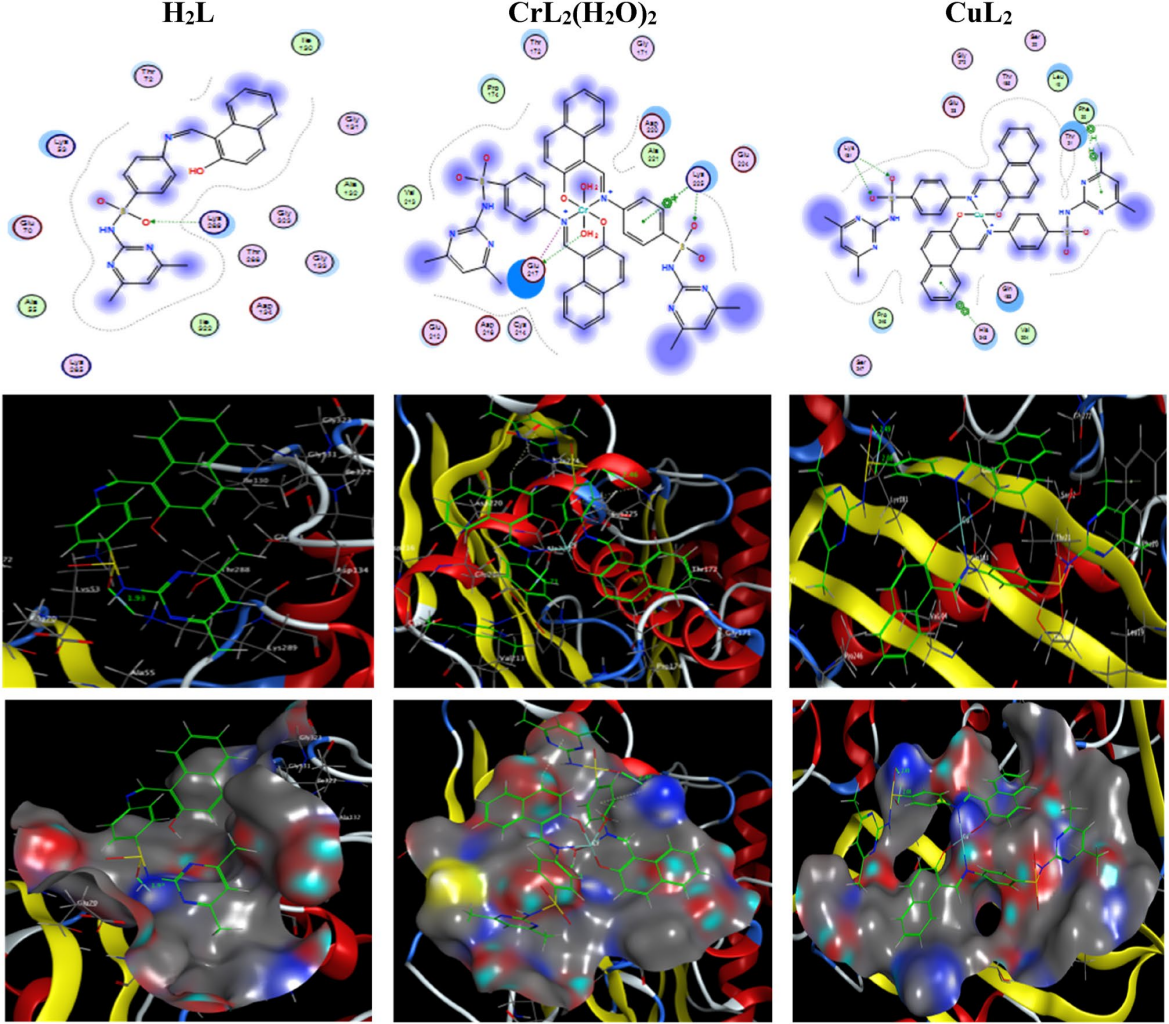
The two- and three-dimensional plots of the interactions between H_2_L, [CrL_2_(H_2_O)_2_].H_2_O and CuL_2_ with the active site of the receptor of liver cancer protein (PDB ID: 5A19).

**Figure 15:**
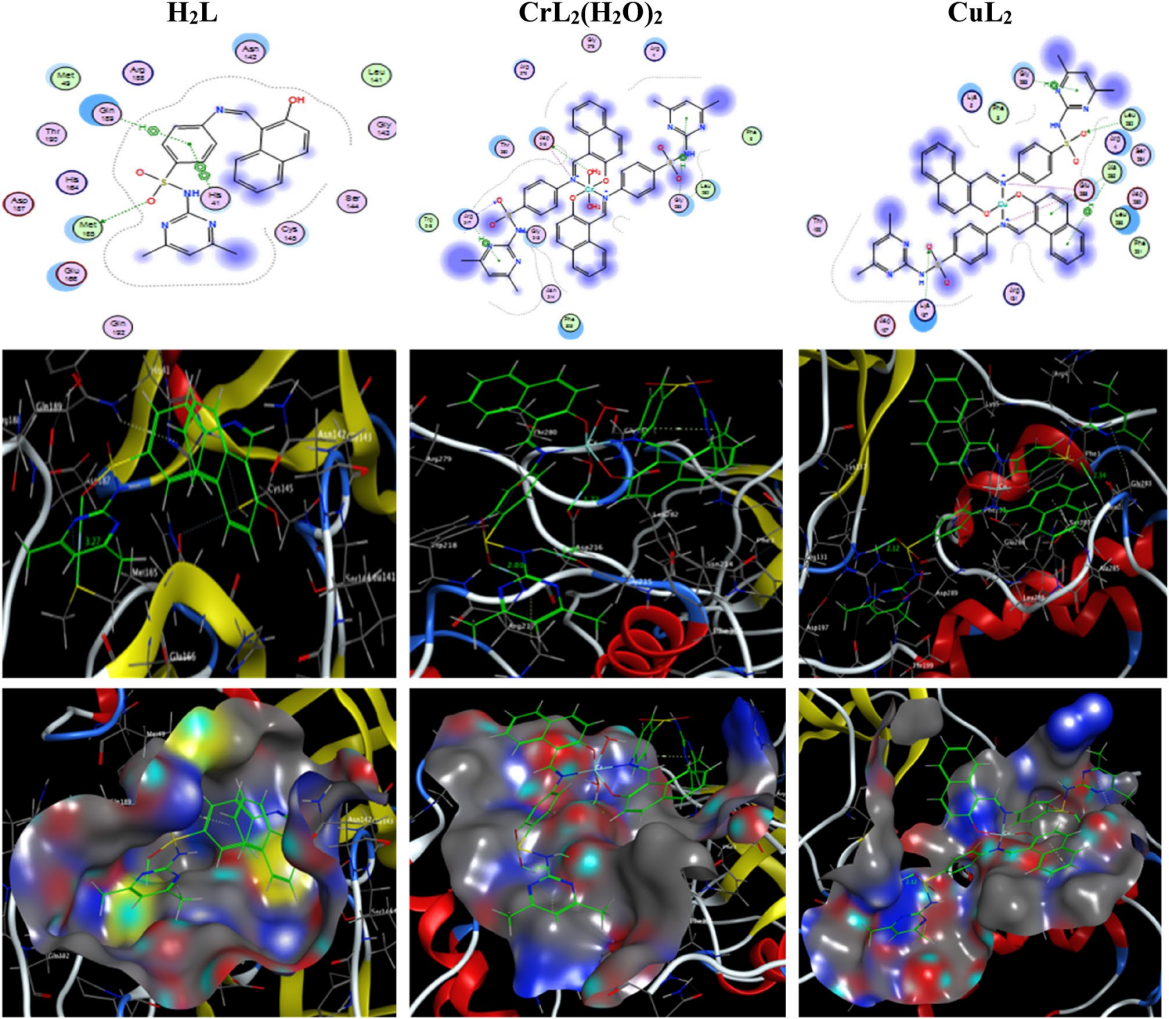
The two- and three-dimensional plots of the interactions between H_2_L, [CrL_2_(H_2_O)_2_].H_2_O and CuL_2_ with the active site of the receptor of COVID-19 main protease viral protein (PDB ID: 6lu7).

The protein-drug affinities could be investigated through molecular docking interaction. The ligand H_2_L and its nano-scale Cu(II) and Cr(II) complexes were docked with protein targets in liver cancer, specifically the receptors of methionine adenosyl-transferases (PDB ID: 5A19) and COVID-19 main protease viral protein (PDB ID: 6lu7). The binding free energies of prepared compounds in case of (PDB ID: 5A19) are − 26.0, − 28.0, and − 39.0 kcal/mol while in case of (PDB ID: 6lu7) are − 8.0, − 23.3 and − 44.1 kcal/mol for H_2_L, CuL_2_ and [CrL_2_(H_2_O)_2_].H_2_O; respectively, (Tables 11 and 12). The stronger interaction reflects more negative binding energy. Hence, molecular interaction data show the highest binding affinity of the [CrL_2_(H_2_O)_2_].H_2_O towards both receptors relative to other compounds. The two- and three-dimensional plots of representative docked structures of H_2_L, [CrL_2_(H_2_O)_2_].H_2_O and CuL_2_ with the receptor’s active site of the liver cancer protein (PDB ID: 5A19) and COVID-19 main protease viral protein (PDB ID: 6lu7) were depicted in Figs. 14 and 15, respectively.

**Table 11:** The Docking interactions found for H_2_L, [CrL_2_(H_2_O)_2_].H_2_O and CuL_2_ with the active sites of the receptor of liver cancer protein (PDB ID: 5A19).

	Receptor	Interaction	Distance(Å)*	E (kcal/mol)
H_2_L				
O 12	NZ LYS 289	H-acceptor	2.82 (1.93)	− 26.0
[CrL_2_(H_2_O)_2_].H_2_O				
O 71	OE2 GLU 217	H-donor	2.66 (1.71)	− 20.9
O 12	NZ LYS 225	H-acceptor	3.25 (2.46)	− 5.6
N 50	OE1 GLU 217	Ionic	3.92	− 0.7
N 50	OE2 GLU 217	Ionic	3.19	− 3.3
O 71	OE2 GLU 217	Ionic	2.66	− 7.2
6-ring	CD LYS 225	pi–H	4.24	− 0.8
6-ring	NZ LYS 225	pi-cation	4.62	− 0.5
CuL_2_				
O 56	NZ LYS 181	H-acceptor	3.08 (2.08)	− 24.3
O 57	NZ LYS 181	H-acceptor	3.16 (2.49)	− 2.4
C 8	6-ring PHE 20	H–pi	3.83	− 0.7
6-ring	CA PHE 20	pi–H	4.14	− 0.5
6-ring	5-ring HIS 243	pi–pi	3.85	− 0.1

*The lengths of H-bonds are in brackets.

**Table 12:** The Docking interactions found for H_2_L, CrL_2_(H_2_O)_2_ and CuL_2_ with the active sites of the receptor of COVID-19 main protease viral protein (PDB ID: 6lu7).

	Receptor	Interaction	Distance(Å)*	E (kcal/mol)
H_2_L				
O 12	SD MET 165	H-donor	3.27 (2.07)	− 6.9
6-ring	NE2 GLN 189	pi–H	4.67	− 1.1
6-ring	5-ring HIS 41	pi–pi	3.98	− 0.0
[CrL_2_(H_2_O)_2_].H_2_O				
N 9	O GLY 215	H-donor	3.00 (2.05)	− 0.7
O 71	OD1 ASP 216	H-donor	2.67 (1.72)	− 18.9
O 12	N ARG 217	H-acceptor	2.90 (2.01)	− 9.4
N 19	OD1 ASP 216	Ionic	3.27	− 2.9
O 71	OD1 ASP 216	Ionic	2.67	− 7.1
O 71	OD2 ASP 216	Ionic	3.18	− 3.4
6-ring	CD ARG 217	pi–H	3.76	− 0.5
6-ring	CA GLY 283	pi–H	4.40	− 1.2
CuL_2_				
O 13	CB LEU 282	H-acceptor	3.42 (2.34)	− 0.9
O 56	NZ LYS 137	H-acceptor	2.95 (2.12)	− 17.2
N 19	OE1 GLU 288	Ionic	3.82	− 0.9
N 19	OE2 GLU 288	Ionic	3.89	− 0.7
N 50	OE2 GLU 288	Ionic	3.82	− 0.9
6-ring	CA GLY 283	pi–H	4.64	− 0.5
6-ring	N ALA 285	pi–H	4.23	− 0.8
6-ring	N ALA 285	pi–H	4.07	− 1.4

*The lengths of H-bonds are in brackets.

## Conclusion

In this study, a novel ligand was synthesized from sulfadimidine sodium and 2-hydroxy-1-naphthaldehyde and coordinated with Cr(II) and Cu(II) to produce novel micro- and nano-complexes that were characterized by various physicochemical and spectral analyses. XRD and TEM results revealed that the nano-sized Cr and Cu complexes were found to be in the sub-nano scale, and temperature variations have a significant impact on the size of the nano complexes. The obtained results from the theoretical study are in good agreement with the experimental results. The antitumor studies on Cr and Cu nanocomplexes showed an inhibition of HEPG-2 cell line and the antitumor activity of the nanocomplexes was improved after heating them at 200 °C. Additionally, the results obtained indicate the Cu nanocomplex showed good DNA cleavage. Furthermore, a molecular docking study revealed that Cr nanocomplex has the highest activity with the receptor of (PDB ID: 5A19) and (PDB ID: 6lu7) in liver cancer and COVID-19, respectively.

## Supplementary Information

Supplementary Information.

## Data Availability

The data used to support the findings of this study are included in the article.
