# Supplementary material for: Synthesis of a new sulfadimidine Schiff base and their nano complexes as potential anti-COVID-19 and anti-cancer activity
Source: Sci Rep. 2023 Jan 27;13:1502. doi: 10.1038/s41598-023-28402-9 (PMC9880939; doi:10.1038/s41598-023-28402-9)
Supplement: Supplementary file 1 — Supplementary Information. [file 41598_2023_28402_MOESM1_ESM.docx]

**Synthesis of a New Sulfadimidine Schiff Base and their Nano Complexes as Potential Anti-COVID-19 and Anti-cancer Activity**

Shimaa Hosny ^1*^, Mona S. Ragab^2^ and Randa F. Abd El-Baki^1^

^1^Department of Chemistry, Faculty of Science, New Valley University, Alkharga 72511, Egypt

^2^Department of Chemistry, Faculty of Science, Cairo University, Giza, 12613, Egypt

*Corresponding author E-mail address: [hosny_shymaa@yahoo.com](mailto:hosny_shymaa@yahoo.com) or [shimaahosny@sci.nvu.edu.eg](mailto:shimaahosny@sci.nvu.edu.eg)

**
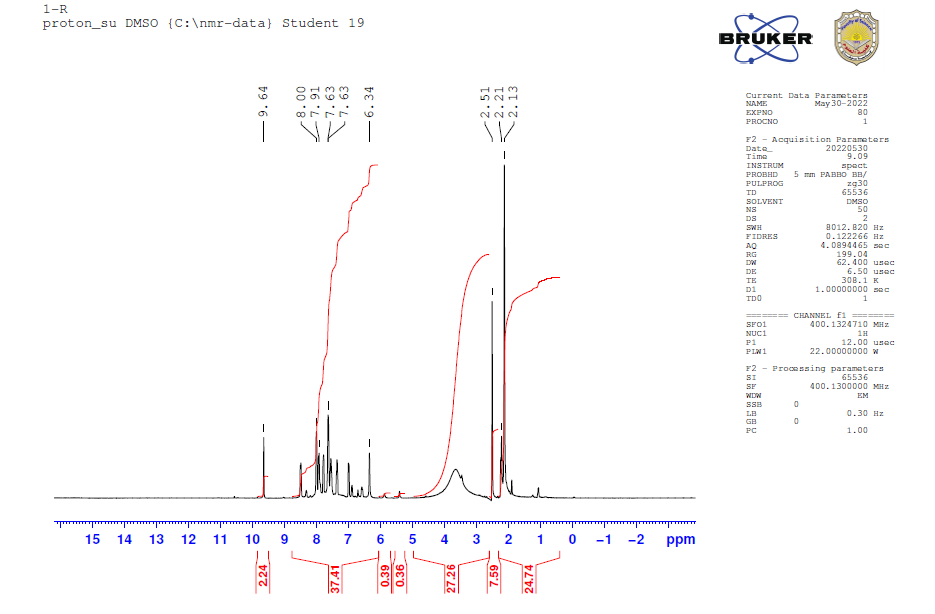
**

**Figure S1.** ^1^H NMR spectrum of H_2_L in DMSO


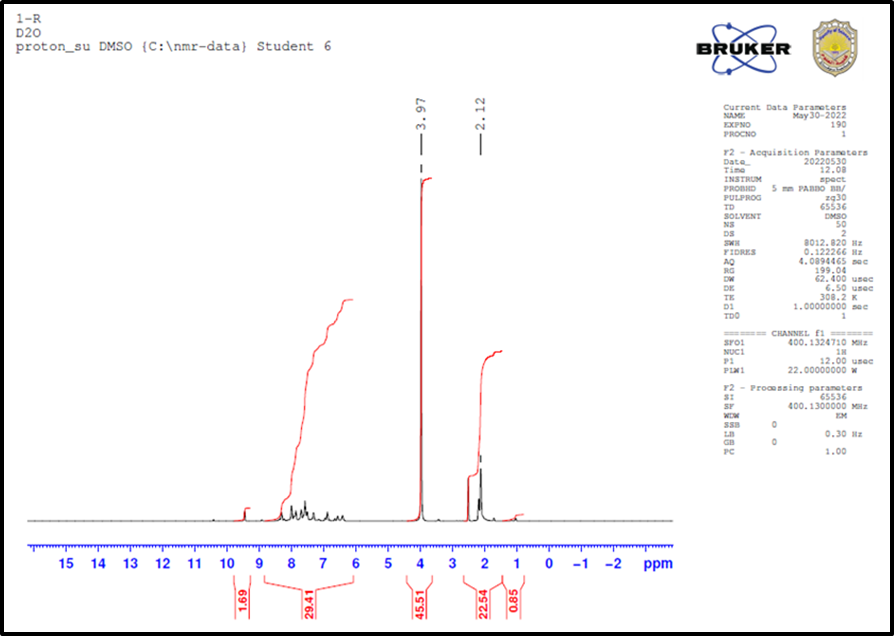


**Figure S2.** ^1^H NMR spectrum of H_2_L in DMSO + D_2_O

**Figure** **S3.** ESI-MS spectra of the H_2_L Schiff base ligand (**A**), Cr(II) complex (**B**), Cu(II) complex (**C**).

**Figure S4.** TG-DTG curves of [CrL_2_(H_2_O)_2_].H_2_O (A) and CuL_2_ (B) micro-complexes. TG-DTG curves of [CrL_2_(H_2_O)_2_].H_2_O (C and E) and CuL_2_ (D and F) nano-complexes before and after heating, respectively.





**Figure S5.** Electronic spectra of H_2_L, its micro and nano-complexes in DMF.

**
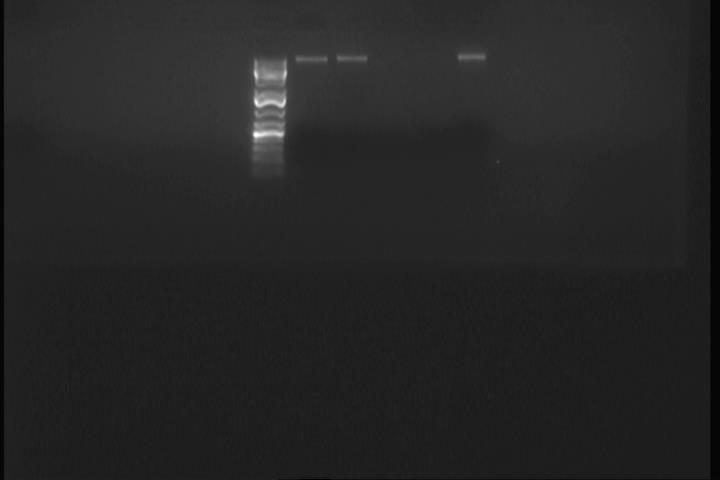
**

**Figure** **S6**. The pattern of DNA binding of the agarose gel electrophoresis diagram showing lane L- marker 1kb DNA Ladder, lane 1 DNA control, lane 2 DNA+DMSO, lane 3: 400 ng DNA+2 mg\ml of Complex, lane 4: 400 ng DNA+1 mg\ml of Complex, lane 5: 400ng DNA+0.5 mg\ml of Complex.


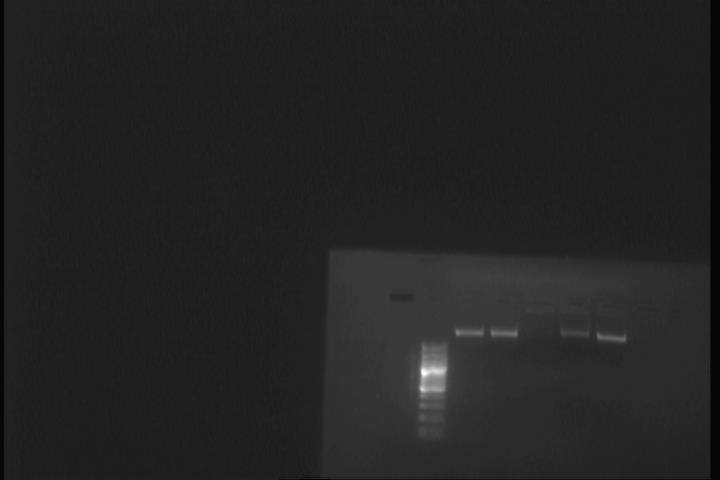
**
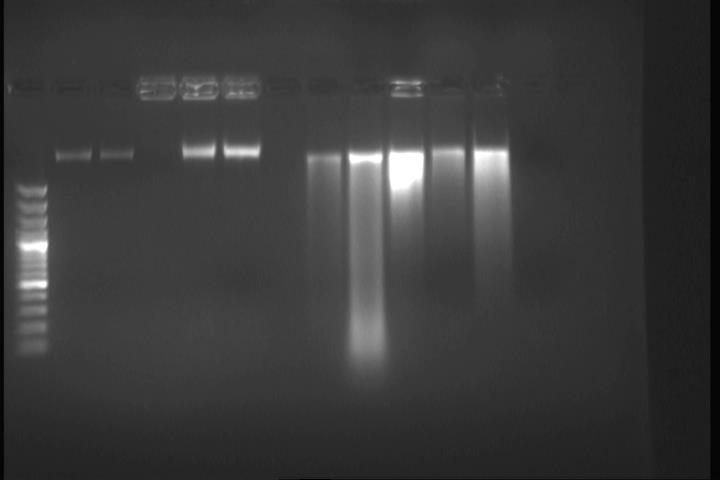
**

**Figure** **S7**. The pattern of DNA binding of the agarose gel electrophoresis diagram showing lane L- marker 1kb DNA Ladder, lane 1: DNA control, lane 2: DNA+DMSO, lane 3: 200 ng DNA+1 mg\ml of Complex, lane 4: 400 ng DNA+1 mg\ml of Complex, lane 5: 800 ng DNA+1 mg\ml of Complex.
